# A comprehensive review on frontotemporal dementia: its impact on language, speech and behavior

**DOI:** 10.1590/1980-5764-DN-2023-0072

**Published:** 2024-04-22

**Authors:** Sha Abbas Mollah, Aditya Nayak, Swapna Barhai, Uddip Maity

**Affiliations:** 1 Rajiv Gandhi University, Health Sciences Karnataka, East West College of Pharmacy, Department of Pharmacy Practice - Bengaluru Karnataka, India.

**Keywords:** Frontotemporal Dementia, Behavior, Alzheimer Disease, Motor Neuron Disease, Speech, Language and Hearing Sciences, Demência Frontotemporal, Comportamento, Doença de Alzheimer, Doença dos Neurônios Motores, Fonoaudiologia

## Abstract

Frontotemporal dementia (FTD) is a progressive neurodegenerative disorder defined by the selective deterioration of the frontal and temporal lobes of the brain. This comprehensive review explored the effect of FTD on language, speech, and behavior. Early symptoms include difficulty in word finding, reduced speech output, and comprehension deficits, often leading to aphasia. The study discussed the profound behavioral changes observed in FTD patients, including apathy, disinhibition, compulsive behaviors, and loss of empathy, the importance of an accurate and early diagnosis, and its challenges. We even reviewed the potential for targeted therapies and the essential role of multidisciplinary care in managing the language, speech, and behavioral aspects of FTD. By examining objective data and comprehensive research on the subject, this study offers valuable insights into FTD’s profound effects on language, speech, and behavior, assisting in improved clinical management and potential therapeutic strategies for this devastating condition.

## RESUMO.

A demência frontotemporal (DFT) é uma doença neurodegenerativa progressiva definida pela deterioração seletiva dos lobos frontal e temporal do cérebro. Esta revisão abrangente explorou o efeito da DFT na linguagem, na fala e no comportamento. Os primeiros sintomas incluem dificuldade em encontrar palavras, redução da produção de fala e déficits de compreensão, muitas vezes levando à afasia. A revisão discutiu as profundas mudanças comportamentais observadas em pacientes com DFT, incluindo apatia, desinibição, comportamentos compulsivos e perda de empatia, a importância de um diagnóstico preciso e precoce e seus desafios. Também foi revisado o potencial para terapias direcionadas e o papel essencial do cuidado multidisciplinar no gerenciamento dos aspectos de linguagem, fala e comportamentais da DFT. Ao examinar os dados objetivos e a pesquisa abrangente sobre o assunto, esta revisão oferece *insights* valiosos sobre os efeitos profundos da DFT na linguagem, na fala e no comportamento, auxiliando no melhor manejo clínico e em possíveis estratégias terapêuticas para essa condição devastadora.

## INTRODUCTION

Frontotemporal dementia (FTD) is a gradually progressing neurodegenerative disorder that impacts the frontal and temporal lobes^1^, responsible for personality, behavior, and language. Common symptoms include changes in behavior, language difficulties, problems planning and organizing tasks, loss of interest in day-to-day activities, and memory issues. FTD is often misdiagnosed as a psychiatric disorder or Alzheimer’s disease (AD)^2^. It is classified into three main subtypes based on its predominant symptoms: behavioral variant FTD (bvFTD), which primarily manifests as alterations in behavior and personality; semantic variant primary progressive aphasia (svPPA), described by language deficits and impaired word formulation and understanding; and non-fluent variant primary progressive aphasia (nfvPPA), associated with difficulty in speech production and language formulation^1^. FTD is more common in men, most prevalent in individuals aged 45 to 65 years^3^, but can occur at any age. Life expectancy after diagnosis is between 6 and 11 years^2^. A blend of genetic predisposition and external influences contributes to its development, requiring a comprehensive diagnostic process encompassing various assessments. Although a definitive remedy remains elusive, therapies can effectively mitigate its symptoms. Swift assessment and intervention in the initial stages are pivotal to enhancing the well-being of individuals affected by FTD^3^.

### Function of the frontal and temporal lobes

About 30% of the brain’s cortical surface is constituted by the frontal lobes, which involve both the prefrontal cortex and regions responsible for motor functions. They play a crucial role in various advanced cognitive functions like planning, decision-making, regulating rewards, understanding social situations, and processing language. Moreover, they are responsible for starting and controlling physical movements^4^, whereas the dorsolateral region of the prefrontal cortex is responsible for executive functions. These functions include remembering information temporarily, cognitive flexibility (adapting to new situations), planning, paying attention, setting goals, and making decisions based on those goals^5^. The frontotemporal networks comprise the Broca’s area located in the inferior frontal gyrus, along with the Wernicke’s area situated in the superior temporal gyrus. These regions are crucial for language processing and additionally contribute to the regulation of behavior. When these networks are damaged, it can lead to problems in understanding and using language, as well as difficulties in controlling impulsive behaviors^6^ ^,^ ^7^. The temporal lobe has two different parts: the laterobasal cortex and the medial temporal lobe. The medial temporal lobe constitutes a cluster of vital structures for episodic memory, encompassing the hippocampal area, perirhinal and entorhinal regions, and the parahippocampal cortices^8^. The anterior temporal lobe is linked to the inferior frontal area and the systems responsible for motor speech output. All these networks play a crucial role in comprehending and employing language, recollecting word meanings and ideas, and grasping social concepts^9^.

### Impact of frontotemporal dementia on language and speech

Many people with motor neuron disease (MND) experience cognitive and behavioral problems. Some of them also meet the diagnostic requirements for frontotemporal dementia, known as FTD-MND^10^. Studies often focus on the connection to the behavioral variant FTD, showing changes in behavior as well as executive function issues^11^ ^,^ ^12^. Not many studies have looked at the language problems in FTD-MND while comparing them with other forms of dementia like PNFA (progressive non-fluent aphasia) or SD (semantic dementia). Previous research mostly compared FTD-MND to bvFTD regarding the occurrence of language issues, without taking into consideration the severity they exhibited^13^. Some studies found that language impairment in FTD-MND is highly prevalent and comparable in severity to language phenotypes observed in FTD. It revealed two distinct language impairment patterns within FTD-MND, with one subgroup showing predominantly syntactic comprehension disturbances, potentially linked to specific brain regions like the left putamen and bilateral caudate^14^ ^,^ ^15^. Another study highlighted that language deficits are common in familial bvFTD and involve significant atrophy in the brain regions responsible for language processing^16^. BvFTD patients eventually develop language deficits and may progress to mutism, similar to PPA. Whereas PPA patients may be initially fluent, increased word-finding difficulty disrupts fluency over time, but fluency dimension must not be used as a defining attribute^17^. Thus, a deeper understanding of language constitution in different genetic subcategories of bvFTD can aid in better patient stratification and disease progression monitoring in future clinical trials.

### Behavior and personality changes

FTD stands as the prevailing clinical syndrome among frontotemporal lobar degeneration cases, connected with pathologies other than AD. The prominent attributes of FTD encompass substantial shifts in character and social behavior, constituting fundamental and supplementary symptoms outlined in diagnostic benchmarks^18^. FTD gives rise to changes in behavior, including a decrease in social interactions, challenges in managing personal behavior, reduced emotional responsiveness, diminished self-awareness, deterioration in hygiene and self-care, cognitive inflexibility, susceptibility to distraction, difficulty in maintaining focus, increased oral consumption, alterations in dietary preferences, repetitive and unvarying actions, as well as compulsive utilization of objects^19^. Individuals afflicted by dementia resulting from subcortical vascular disease might exhibit certain behavioral traits resembling those found in FTD, owing to the robust interlinkages between subcortical white matter and the frontal lobes^20^. Studies that compare behavior in FTD with other forms of dementia have been carried out; however, these studies frequently fall short in thoroughly investigating the atypical behaviors characteristic of FTD. The behavioral attributes outlined in the agreed-upon criteria for diagnosing FTD are wide-ranging, encompassing a variety of potential behavioral modifications. As a result, the exact nature of behavioral shifts within a group of FTD individuals remains ambiguous^21^ ^,^ ^22^. The characteristics that most effectively differentiate FTD from other types of dementia can be categorized into three primary domains:

- Affect (loss of basic emotions);
- Oral behaviors (overeating, food cramming), and;
- Repetitive, stereotyped behaviors (motor and verbal stereotypies, pacing).

The exact mechanisms underlying patients’ unusual behaviors and the role of specific brain structures in contributing to these changes require further exploration^23^. According to other researchers, FTD can be categorized into two contrasting subsyndromes: FTD-A (characterized by apathy) and FTD-D (marked by disinhibition). FTD-A is linked to widespread atrophy of the frontal lobes that extends into the dorsolateral frontal cortex, resulting in apathy, inertia, and a decline in motivation. FTD-D is associated with atrophy of the orbitomedial frontal lobes and temporal pole, leading to disinhibition, a tendency to become easily distracted, and purposeless excessive activity. While both FTD-A and FTD-D display significant behavioral disruptions, they also show noteworthy retention of practical skills. Individuals with these syndromes do not primarily exhibit aphasic symptoms or encounter deficits in perceptual or spatial functions^24^. Understanding the distinct behavioral profiles in frontotemporal dementia, including eating behavior, is crucial for accurate diagnosis and appropriate management of patients, as these symptoms can be among the earliest signs of the disease. Differences in eating behavior patterns among varied types of dementia can also provide valuable insights into the underlying neurological mechanisms involved in each condition^25^.

### Functional impairment

FTD is a brain disease that usually starts during adulthood when people are still working and supporting their families. It leads to gradual changes in daily activities, but the rate at which these changes occur is not well understood. This condition affects thinking and behavioral patterns, making it hard for individuals to perform their usual roles in life, such as being parents, working, and maintaining relationships with others^26^ ^,^ ^27^. Studies examined how daily abilities change over time in various types of FTD using a suitable tool. The results showed that patients with bvFTD pathological, SD, and PNFA variants of FTD experienced a significant decline in their daily activities, except for those identified as bvFTD phenocopy patients^28^. The Disability Assessment for Dementia (DAD) scale is a tool used for evaluating functional impairment in individuals with the disease, including FTD. The DAD assesses both basic activities of daily living (BADL) and instrumental activities of daily living (IADL). Monitoring functional impairment can also help predict how the disease may progress over time. Researchers are studying these factors to develop better ways to care for individuals with bvFTD and improve their quality of life^29^.

### Quality of life and caregiver burden

FTD, broadly young-onset dementia (YOD) is often misdiagnosed or underdiagnosed due to atypical symptoms, such as personality and changes in behavior or language deficits, which may be attributed to stress or depression^30^. YOD has a more aggressive course than late-onset dementia (LOD), with more steep decline and decreased survival. It can have a significant impact on the quality of life (QOL) of both patients and their families. Individuals afflicted with YOD undergo a gradual decline in activities of daily living, leading to a loss of independence and accompanying neuropsychiatric comorbidities, significantly impacting their QOL^31^ ^,^ ^32^. QOL within the context of dementia is a multi-dimensional concept that encompasses psychological contentment, the subjective perception of QOL, adeptness in behavior, and tangible surroundings. Individuals with YOD often sustain residence within their communities for extended periods, leaning on familial assistance in an informal capacity. This inclination might result in inadequate utilization of professional healthcare resources and place extra stress on the family unit^33^.

BvFTD impacts a considerable population, often manifesting at a younger age compared to AD, thereby introducing distinct difficulties for both patients and caregivers. Those caring for individuals with FTD might encounter reduced health-related QOL and elevated distress levels in contrast to caregivers of those with AD^34^. FTD caregivers experience a significant burden due to the pronounced and challenging behavioral problems associated with FTD and it is not as well-known as AD, leading to insufficient comprehension and backing from the community and the healthcare system^35^. Caring for patients with bvFTD can be more challenging for caregivers because there is often less support and awareness about this condition compared to AD. Caregivers of patients with both bvFTD and AD, who have been diagnosed more recently, tend to feel more affected in their overall QOL. They experience a higher emotional burden, especially due to neuropsychiatric symptoms, but when considering all emotional burdens, there is no significant difference between bvFTD and AD caregivers^36^. Depression and neuropsychiatric symptoms are important predictors of reduced QOL in individuals residing within the community and those in institutional care with LOD, but there are limited studies on QOL in YOD^33^.

## DIAGNOSIS

BvFTD is a neurological condition in which patients experience subtle and gradual alterations in their personality and social behavior. These changes signal the progressive breakdown of neural circuits responsible for social cognition, emotional control, motivation, and decision-making. The disease’s underlying pathological changes are diverse and marked by different types of abnormal intraneuronal inclusions^37^. These diverse presentations of FTD pose unique diagnostic challenges for clinicians. Distinguishing FTD from other forms of dementia and psychiatric disorders with overlapping symptoms can be difficult, leading to potential delays in diagnosis^38^.

FTD presents behavioral changes while overall cognitive abilities remain relatively stable. Tests for verbal fluency, memory, and language are reliable tools to track the disease’s progression^39^. FTD is mostly diagnosed through clinical assessment. Although there may be challenges in diagnosing the condition, the presence of abnormal social behavior, changes in eating habits, repetitive behaviors, and apathy without significant memory or visuospatial problems typically points to the correct diagnosis^40^.

Accurate and early diagnosis of FTD is crucial for appropriate patient care. Delayed diagnosis may lead to missed opportunities for early interventions and appropriate symptoms management. Timely diagnosis provides caregivers with a clearer understanding of the clinical status and prognosis of the patient, enabling them to plan and provide proper care and support. Neuropsychiatric symptom measures can be useful tools in the diagnostic process, helping to identify specific behavioral changes associated with FTD. However, more sensitive assessments are required to delineate the precise behavioral and linguistic deficits specific to FTD, which can aid in distinguishing it from other conditions. Magnetic resonance imaging, single photon emission computed tomography, or computed tomography scans were obtained for all patients during their initial visit and subsequently if possible. Language difficulties and social behavioral changes were particularly important in differentiating FTD from other conditions. A thorough clinical assessment, encompassing evaluations of language, socio-emotional capabilities, cognition, and neuroimaging, is essential for an accurate diagnosis of FTD^41^ ^,^ ^42^ ^,^ ^43^ ^,^ ^44^.

## MANAGEMENT

Treating FTD requires a personalized approach. As of now, no treatment can prevent the advancement of the neurodegenerative disease. The primary objective of medical therapy is to provide relief from symptoms and improve the patient’s comfort^45^. At present, there are no treatments approved by the Food and Drug Administration (FDA) agency specifically for FTD. Nevertheless, certain selective serotonin receptor inhibitors (SSRIs) such as paroxetine, sertraline, or fluoxetine can be employed to mitigate repetitive actions, disinhibition-impulsivity, and eating disorders in individuals with FTD. In cases of notable disruptions or heightened agitation, modest doses of trazodone or an atypical antipsychotic like aripiprazole can aid in the management of such behaviors among those affected by FTD^46^. Cholinesterase inhibitors and N-methyl-D-aspartic acid (NMDA) receptor antagonists find utility in the pharmacological management of behavioral manifestations. Similarly, selective serotonin reuptake inhibitors and analogous treatments, including antipsychotics, dopaminergic therapies, anti-epileptic agents, and oxytocin, are applied to address motor symptoms. Tetrabenazine has demonstrated positive effects in addressing severe tics and stereotypies in FTD cases. However, the use of cholinergic medications like donepezil exacerbated symptoms, and cessation led to an improvement in behavioral symptoms and a reduction in caregiver burden. N-methyl-D-aspartate receptor inhibitors exhibited mixed outcomes in trials, although more recent studies indicate enhanced behavioral symptom alleviation in select instances of moderate-to-severe bvFTD^47^ ^,^ ^48^. According to a study, at least half of the subjects who previously experienced depressive symptoms, carbohydrate craving, disinhibition, and compulsions showed improvement in these symptoms after receiving treatment with SSRIs^49^.

Nonpharmacological management of FTD includes education, behavioral interventions, and support for caregivers^46^. In addressing the described syndromes, a comprehensive approach involves engaging in occupational therapy, physical therapy, and speech/swallow therapy. Physical therapy places emphasis on enhancing gait and balance to avoid falls and enhance mobility, potentially requiring the use of gait aids. Occupational therapists assess household safety to mitigate the risk of falls and provide support in daily tasks for individuals contending with fine motor manipulation difficulties or apraxia. Speech therapy, particularly under the guidance of therapists skilled in handling neurodegenerative aphasias, proves advantageous for those affected by PPA. Additionally, it can be beneficial for individuals within the FTD spectrum who encounter challenges like dysarthria, apraxia, or hypophonia^47^.

Promising clinical trials are currently in progress for FTD targeting TDP-43 and tau, investigating treatment approaches including tau vaccines, inhibitors of tau phosphorylation and acetylation, tau-antibodies, and agents that stabilize microtubules. Meanwhile, there is active exploration and development going on for gene-editing therapies that employ antisense oligonucleotides to decrease the expansion of C9 or f72 or elevate progranulin (prgn) expression in genetic FTD^48^.

In conclusion, FTD is a complex and gradually progressive neurodegenerative disorder that affects primarily the frontal and temporal lobes, leading to profound changes in behavior, personality, and language. It is often misdiagnosed due to its atypical symptoms, which can overlap with psychiatric disorders or AD. FTD is classified into subgroups based on predominant symptoms, including svPPA, nfvPPA, and bvFTD. The impact of FTD on language and speech is significant, with distinct patterns of language impairment observed in different genetic subtypes. Moreover, FTD is associated with substantial functional impairment, affecting daily activities and QOL for both patients and their caregivers. Timely and accurate diagnosis is crucial for appropriate management and support, as there are currently no disease-modifying treatments available. Non-pharmacological interventions, behavioral therapy, and caregiver support play an essential role in managing the symptoms of FTD. Ongoing research and clinical trials hold promise for future treatments targeting specific underlying pathological changes. Improved understanding and early evaluation of FTD are essential to enhance the QOL of patients and their families dealing with this challenging condition.
